# Analysis of Thermal Imaging Performance under Extreme Foggy Conditions: Applications to Autonomous Driving

**DOI:** 10.3390/jimaging8110306

**Published:** 2022-11-09

**Authors:** Josué Manuel Rivera Velázquez, Louahdi Khoudour, Guillaume Saint Pierre, Pierre Duthon, Sébastien Liandrat, Frédéric Bernardin, Sharon Fiss, Igor Ivanov, Raz Peleg

**Affiliations:** 1 Cerema Occitanie, Research Team “Intelligent Transport Systems”, 1 Avenue du Colonel Roche, 31400 Toulouse, France; 2 Cerema Centre-Est, Research Team “Intelligent Transport Systems”, 8-10, Rue Bernard Palissy, 63017 Clermont-Ferrand, France; 3 ADASKY, 7 Hamada Street, Yokneam Illit 2069206, Israel

**Keywords:** thermal imaging, meterological optical range, object detection, operability limits

## Abstract

Object detection is recognized as one of the most critical research areas for the perception of self-driving cars. Current vision systems combine visible imaging, LIDAR, and/or RADAR technology, allowing perception of the vehicle’s surroundings. However, harsh weather conditions mitigate the performances of these systems. Under these circumstances, thermal imaging becomes the complementary solution to current systems not only because it makes it possible to detect and recognize the environment in the most extreme conditions, but also because thermal images are compatible with detection and recognition algorithms, such as those based on artificial neural networks. In this paper, an analysis of the resilience of thermal sensors in very unfavorable fog conditions is presented. The goal was to study the operational limits, i.e., the very degraded fog situation beyond which a thermal camera becomes unreliable. For the analysis, the mean pixel intensity and the contrast were used as indicators. Results showed that the angle of view (AOV) of a thermal camera is a determining parameter for object detection in foggy conditions. Additionally, results show that cameras with AOVs 18° and 30° are suitable for object detection, even under thick fog conditions (from 13 m meteorological optical range). These results were extended using object detection software, with which it is shown that, for the pedestrian, a detection rate ≥90% was achieved using the images from the 18° and 30° cameras.

## 1. Introduction

Advanced driver assistance systems (ADAS) and obstacle avoidance systems (forward collision warning—FCW and active emergency braking—AEB) are areas of development identified for the transport industry. Even so, statistics show that 1.35 million people die each year on the world’s roads, of which 54% are vulnerable road users [1,2]. Specifically, statistics show that 80% of accidents involving a pedestrian in the USA occur in poor light or weather conditions [1,2]. Consequently, a huge part of the research work within ADAS and obstacle avoidance systems addresses vehicle perception in adverse weather conditions.

Current vision systems combine visible imaging, LIDAR and/or RADAR technology for perception of the vehicle’s surroundings. However, certain factors mitigate the performance of these systems. If the light is too low, the distances for detecting and recognizing obstacles become too short to ensure the safety of the vehicle and its environment. Under these conditions, thermal imaging is the complementary solution because it makes it possible to detect and recognize the environment in the most extreme conditions (night, fog, rain). Thermal imaging applied to autonomous vehicles thus contributes to the complementarity and redundancy of sensors. In addition, thermal images are compatible with detection and recognition algorithms based on artificial intelligence and neural networks [3,4,5].

In this article, an analysis of the performance of thermal cameras under extreme weather conditions is presented. This analysis is part of the work carried out within the European project “All Weather Autonomous Real Logistics Operations and Demonstrations” (AWARD) [6], where the goal is the analysis of different technological solutions for the automation of goods transport vehicles. The presence of adverse weather conditions is the key factor for this study. However, in this article, we only present the results corresponding to the analysis of the thermal sensors concerning the presence of extreme fog conditions. To be more precise, the sensors were subjected to increasingly thick fog conditions, and the objective of the study was to find what is the breaking point, i.e., the very degraded fog situation beyond which the thermal sensor becomes unusable. In light of the results found, we can advise thermal imaging practitioners on the behavior of these sensors in the presence of harsh fog, which is one of the AWARD project’s main goals. Thus, good practices and lessons learned from this study will probably emerge. On the other hand, as it is not possible to have all the desired natural fog conditions in a reasonable period of time, a fog generation platform was used to create levels of fog almost instantaneously. Thus, tests were set up within the Cerema PAVIN Fog and Rain platform [7,8], a short description of which is provided in Section 2.1.

To analyze the responses of the thermal sensors in terms of their ability to distinguish objects in a scene, two scenarios were used:

- The first scenario consisted of evaluating the visibility of four thermal targets (black bodies heated at different temperatures) in fixed static positions inside the PAVIN platform. Two quality indicators of the image extracted from the targets were used: the intensity and the contrast of the targets compared to the background. Other variables were involved in this experiment: the angle of view of the camera (four different angles studied), the density of the fog (given by the meteorological optical range (MOR), as defined in Section 2.1), the distances between the sensors and the targets, and the temperatures of the targets. The aim was to find the limit to the observability (quantified by the contrast) of objects in relation to their temperature.
- The second experiment consisted in analyzing scenes where a car and a pedestrian were present. Objects (car and pedestrian), both in motion and motionless, were studied. As in the first scenario, other variables such as the angle of view of the camera, the density of the fog, and the distances between the sensors and the objects were also analyzed. To assess the observability of the car and the pedestrian, YOLO [9] was used. The goal was to study the possibility of recognizing events as the density of the fog increases. This was again to check the limit of the thermal sensor when it is used to recognize scenarios in very dense fog.

In the literature, there are few papers on object detection by thermal cameras in degraded meteorological conditions. Studies on object detection are mostly only focused on favorable meteorological conditions, or used other sensors, such as lidar and CCTV cameras. For example, this is the case for most datasets [10,11,12]. From time to time, thermal cameras are used as complementary sensors to video or lidar devices, but they have almost never been used alone during degraded conditions.

Regarding the combined use of thermal images and object detection/identification software, some works in the literature present fusions between RGB and thermal representations to improve detection in a Faster-RCNN model [5]. Works that show the implementation of the YOLO model and thermal images for the detection of objects under different adverse meteorological conditions also exist [3,4], although they lack formality regarding the relationship between the detection rate and the meteorological conditions that exist. Unlike what is presented in the literature, our proposal presents a detailed analysis of the response of the detection software (in this case the YOLO model) concerning the intensity of the fog, the angle of view of the camera, and the distance between the camera and the objects.

Concerning degraded meteorological conditions, only some studies exist, but based on lidar sensors [13,14,15,16], visible cameras [17], or by fusion of data [18,19,20].

Other studies focused on the choice of wavelength to use for cameras (visible, near infrared, or thermal infrared) in foggy conditions, but they have not included obstacle detection, such as detection of pedestrians or other objects [21,22]. However, there is no analysis of pedestrian detector by thermal camera in these studies. The novelty of this study is therefore, to propose to analyze the impact of foggy conditions on a thermal camera, to see the resilience of the latter and using a pedestrian detector known from the literature.

This paper is organized as follows: In Section 2, the definitions of the variables, tools, and physical quantities involved in this research work are presented. In Section 3, the experiments performed within the Cerema’s PAVIN Fog and Rain platform using the thermal cameras developed by ADASKY are explained in detail. Then, in Section 4, all the results obtained from the aforementioned experiments are presented. Section 5 contains a summary of the obtained results and the authors’ comments about these. Finally, in Section 6, the conclusions of this research work are presented.

## 2. Definitions, Tools, and Physical Quantities

### 2.1. The PAVIN Fog and Rain Platform

**Figure 1:**
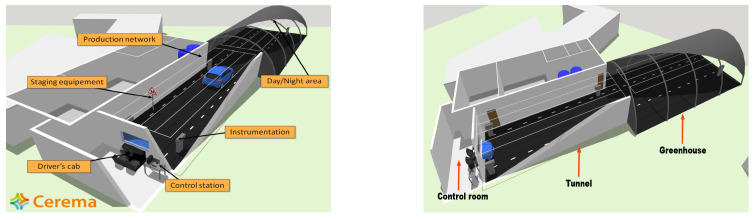
PAVIN fog and Rain platform [7,8].

To test the thermal imaging in front of foggy conditions, experiments were carried out within the Cerema PAVIN Fog and Rain platform [7,8,23], a structure of 30 m long, composed of three sections: (a) a control room, where the computers for the control of the meteorological conditions and the data acquisition by the sensors are located, (b) the tunnel, and (c) greenhouse for day and night conditions. The first 15 m (distance measured from the outside wall of the control room) are part of the closed structure, and the last 15 m are inside the greenhouse, which can be uncovered during the day (see Figure 1).

In this platform, various weather conditions, such as fog and rain, can be reproduced. Moreover, various scene elements can be put in the test chamber to recreate calibrated test scenarios (i.e., reference targets) or real-world scenarios (i.e., road markings, traffic signs, vehicles, and pedestrians). This platform is used in public projects, such as the AWARD project, but also for private testing (confidential renting of the platform). In this study, only fog conditions will be addressed. Fog density is measured by the meterological optical range (MOR), whose definition is given in Section 2.3. In the chamber, the MOR range is of 10 to 1000 m. An MOR of 10 m corresponds to a very dense fog, and an MOR of 1000 m corresponds to an almost clear environment, as is illustrated later in Section 3.

### 2.2. Technical Description of the Cameras

**Figure 2:**
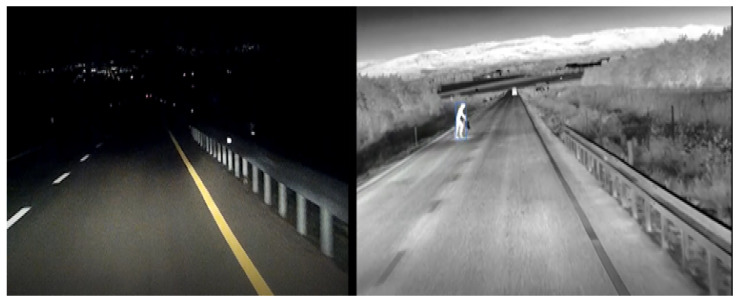
Use of the ADASKY LWIR camera to improve visibility on roads.

ADASKY offers a set of advanced LWIR cameras which enhance ADAS and AEBS capabilities, improving the driver’s safety and the automated control and safety systems of autonomous vehicles, thereby protecting the lives of all road users (see Figure 2). In this research, ADASKY’s automotive LWIR cameras were tested under conditions of extremely low visibility caused by the presence of fog.

**Figure 3:**
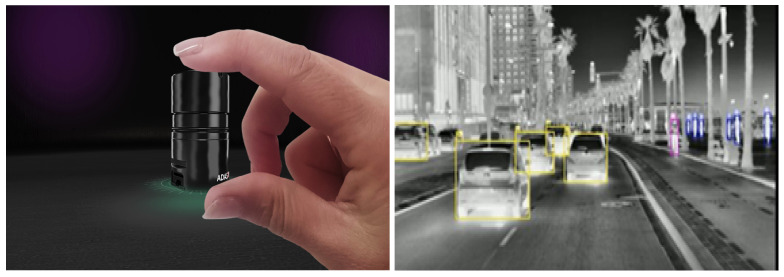
ADASKY’s LWIR camera.

ADASKY’s LWIR camera (illustrated in Figure 3) is based on the company’s proprietary ISP (“ADA1”) designed solely by ADASKY’s engineering team. This ISP chip hosts image enhancement algorithms. One of the objectives of this research work was to assess the limits of the ADASKY sensor under very foggy conditions.

To the best of our knowledge, ADASKY’s camera is the only shutter-less LWIR camera on the market and is thus constantly operational, allowing it to be a legitimate component in autonomous active safety systems. This unique proprietary feature is responsible for the camera’s high image quality, eliminating the need for moving parts and resulting in cost reduction, extended camera life, smaller camera size, and lower power consumption. About this, Table 1 summarizes the technical specifications of ADASKY’s camera.

As a side note—shuttered cameras lose on average 0.5 s of data every 2–3 min for calibration. This implies that the vehicle will “lose its sight” for about 11 m while moving at a speed of 80 KM/H. Moreover, shuttered cameras exhibit image quality degradation between the shutter cycles, whereas ADASKY’s camera maintains high image quality continuously.

ADASKY’s Video Out streams can be broadcast simultaneously in a 14-bit grayscale optimized for computer vision and an 8-bit grayscale optimized for viewing. In this research, only the 8-bit output was used; however, it should be noted that the results presented in this research work could be improved by exploiting the full potential of 14-bit images instead. Such analysis has been left as future work.

ADASKY’s product is also empowered by detection and perception algorithms, which are not part of the AWARD program at this point and thus are not covered in this analysis.

### 2.3. General Definitions

Next, we present the definitions of some concepts used throughout this document.

Target: It is the object of study, used to evaluate the capabilities of thermal cameras. We use the word target to refer to one of thermal blackbodies (calibrated targets used during static scenarios; Section 3.1), a pedestrian, or a car (the last two used during dynamic scenarios, Section 3.2).

Meterological Optical Range (MOR): In [24] m MOR is defined as the length of path in the atmosphere required to reduce the luminous flux in a collimated beam from an incandescent lamp, at a color temperature of 2700 K, to 5 per cent of its original value. In simple words, MOR is the estimation of visibility considering physical factors (luminosity, humidity, etc.). It is related to the intuitive concept of visibility through the contrast threshold. For this study, the term MOR is used to refer to the intensity of fog present in the experimentation platform, and it is measured in meters. Throughout this document, terms MOR and meteorological visibility will be used without distinction to denote this parameter.

Camera-to-target distance: It is the distance (measured in meters) between the thermal cameras and the observed objects, either the thermal targets (in the case of static scenarios) or the pedestrian and car (in the case of dynamic scenarios). Throughout this document, the terms distance and camera-to-target distance will be used without distinction to denote this parameter.

Observability: We use this term to denote the property of a target to be distinguished from the background, either by a human entity or by a piece of detection software. For both cases, and since for this study only grayscale images were used, this parameter is strongly linked to the contrast, i.e., the difference in light intensities between the target and the background.

**Figure 4:**
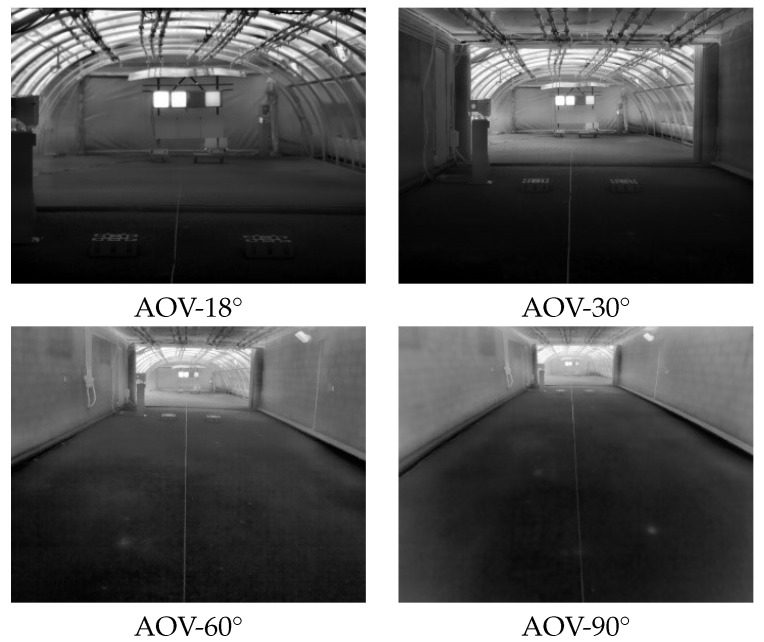
Images from the four cameras used during the tests. The AOVs were: 18°, 30°, 60°, and 90°.

Angle Of View (AOV): It measures the angle of the scene in front of the camera that is captured by the camera’s sensor [25]. For this study, four different thermal cameras with different degrees of AOV were used: 18°, 30°, 60°, and 90°. All cameras were placed at the same distance from the targets. Figure 4 presents examples of the images captured with the four thermal cameras used during the tests. The four cameras were used in parallel to record all the scenarios and protocols. The use of concurrent cameras with different AOVs allowed us to analyze the impact of MOR on a camera’s AOV. In addition to the four thermal cameras, an RGB camera was used to record the scenarios. This camera was placed in the same position (with respect to targets) as the thermal cameras. This RGB camera was used as a reference in certain cases.

## 3. Testing Protocol

The experiments performed within the Cerema’s PAVIN Fog and Rain platform using the thermal cameras developed by ADASKY were carried out over three days. During this time, about 360 min of video were recorded by each camera. The experiments were carried out under different conditions, which constituted two scenarios:

- Static scenarios using four motionless heated blackbodies at different temperatures.
- Dynamic scenarios involving an electric car and a pedestrian, either in motion or motionless.

With the static scenarios, the impacts of adverse meteorological conditions on the observability of the different targets at different temperatures was analyzed. From there, conjectures were established regarding the relationships between the different available variables: MOR, temperature, camera-to-target distance, and AOV. Then, the capabilities of thermal cameras were studied using two indicators: the mean pixel intensity and the contrast of the targets compared to the background. The main aim was to establish the limits of operability of the different cameras according to the intensity of fog.

In the dynamic scenarios, the operability limits obtained during the static tests were confirmed, but this time, using animated objects, such as a pedestrian and a car. Unlike the thermal targets, the temperatures of the pedestrian and the car were not uniform, making it impossible to execute the same analysis carried out for the static tests. To overcome this, object recognition software was used for the assessment of the observability of the pedestrian and the car. For this, the famous YOLO software was used [9,26]—deep learning-based software for object detection and recognition, including pedestrians and cars. To obtain a result that was independent of the recognition method, it was decided to use the YOLOv5s pre-trained model without any modification, only pre-trained by the COCO dataset [27]. Thus, without model training, the recognition rate depends mostly on the contrast level in the images, resulting in the use of the same indicator as in the static scenarios. Hence, the dynamic scenarios extend the results obtained during the static scenarios. Next, both static and dynamic scenarios are described in detail.

### 3.1. Static Tests

**Figure 5:**
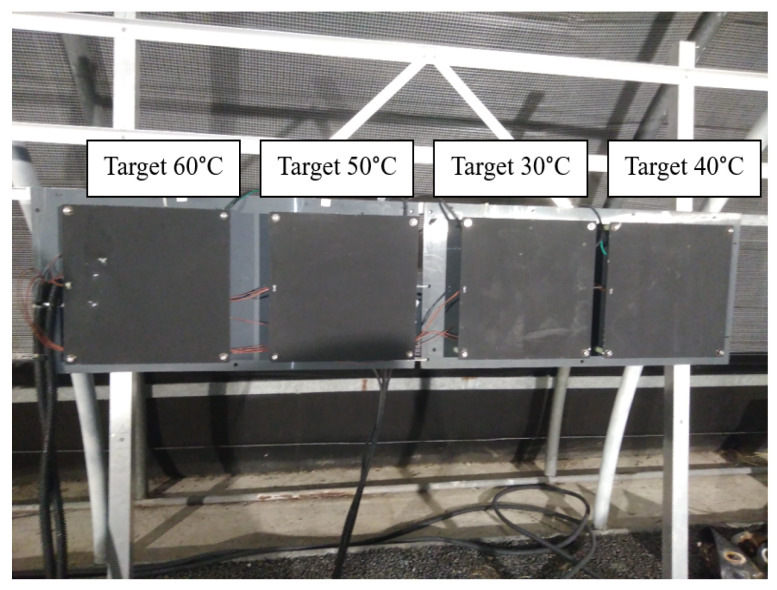
Thermal targets for static scenarios.

The objective of static scenarios was to study the impact of foggy weather conditions on the performance of thermal cameras. For this, four heated blackbodies at different temperatures were used to represent a group of four thermal targets: 30, 40, 50, and 60 °C (see Figure 5).

Such thermal targets are used as references to measure the range of the thermal cameras concerning different MORs.

**Figure 6:**
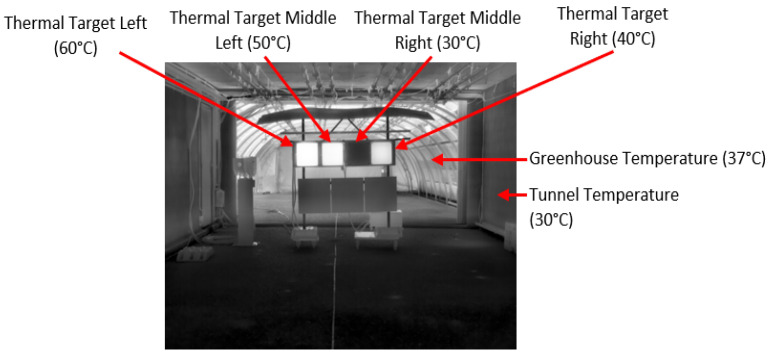
Description of static scenarios.

**Figure 7:**
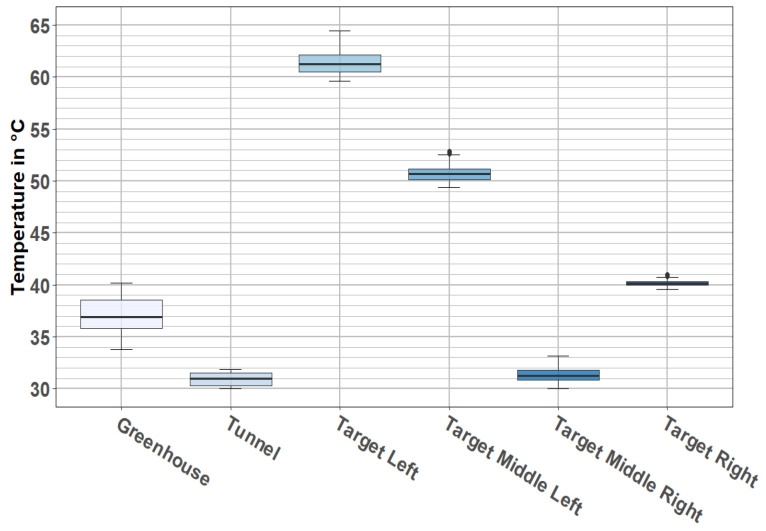
Boxplot of targets’ and rooms’ temperatures during static tests.

The view perceived by thermal cameras is illustrated in Figure 6. As it is shown, cameras perceived the four thermal targets and the two structures that were part of the PAVIN platform (tunnel and greenhouse). These two structures were at different temperatures: the closed section or tunnel had an average temperature of 30 °C, and the open section or greenhouse had an average temperature of 37 °C (see Figure 7). Figure 7 shows a relative stability of temperatures for each target, as all the temperatures were concentrated around the median value, and this was true for every location.

For the study of the static scenarios, about 320 min of video (80 min of data from each camera) were analyzed. These data were classified into two groups according to the definition of fog given by national meteorological service in UK [28]: fog is essentially a cloud at ground level that causes a reduction in visibility to less than 1000 m; it must be much thicker for the visibility to drop below 180 m. Severe disruption to transport occurs when the visibility falls to below 50 m over a wide area (referred to as dense fog). Thus, recorded data are classified into the following groups:

- Use case (a)—Reference data. Videos recorded without the presence of fog (MOR above 1000 m);
- Use case (b)—Data from foggy weather. Videos recorded with a MOR (continuous variable) varied from an initial value of 10 m (denser fog) to 160 m (dissipated fog).

**Figure 8:**
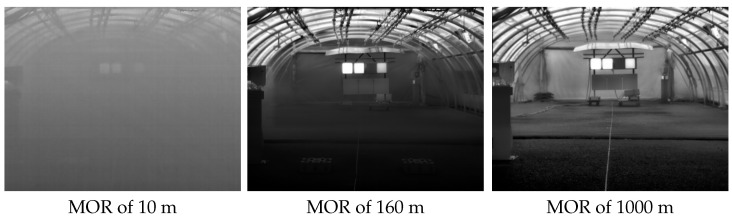
Examples of different MORs.

Figure 8 shows an example of each of these two groups. On the left side there is an image with visibility of 10 m, and the image in the middle corresponds to visibility of 160 m; both images come from a video belonging to the use case (b). The image on the right corresponds to the reference data (use case (a)), where the MOR is around 1000 m.

As this study was aimed at analyzing the capabilities of the thermal cameras under foggy weather conditions, most of the analysis was oriented toward the images from the use case (b). Thus, unless otherwise indicated, from now on, the presented results belong to the videos from use case (b).

**Figure 9:**
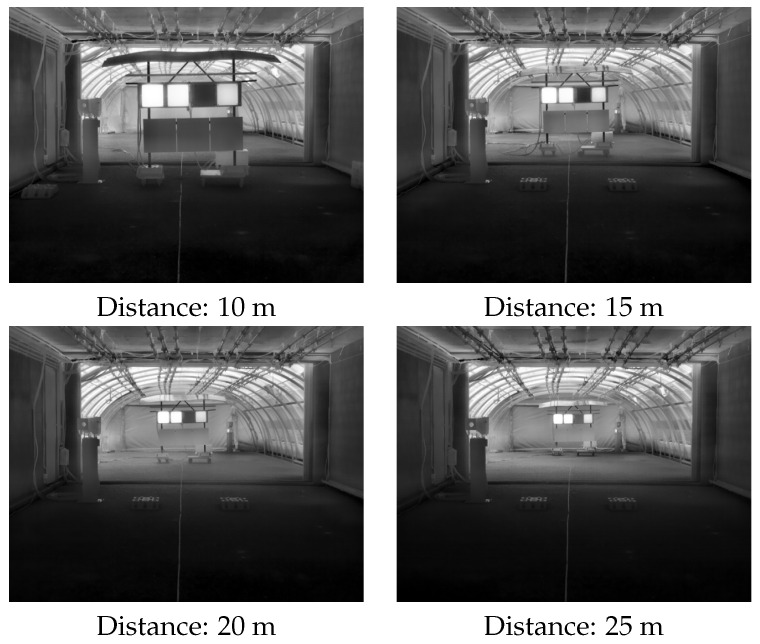
The four different camera-to-target distances used during static scenarios.

The impact of the camera-to-target distance is also included in this study. For this, four fixed positions were used: 10, 15, 20, and 25 m. Figure 9 illustrates the perceptions of these four distances by the AOV 30° camera.

### 3.2. Dynamic Tests

**Figure 10:**
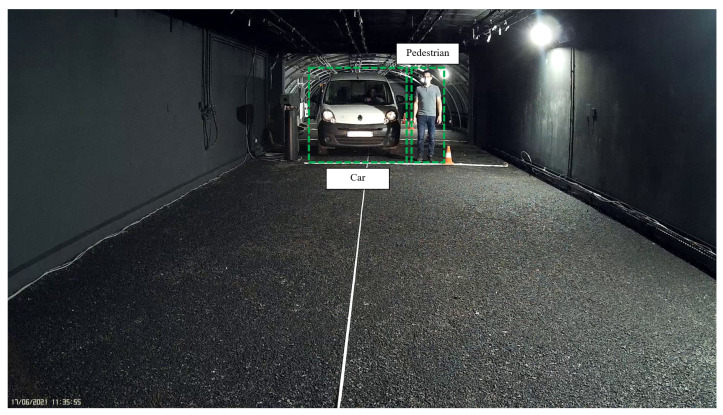
Description of dynamic scenarios.

As in the static scenarios, the objective of the dynamic scenarios was to analyze the impact of foggy weather conditions in the operability limits of thermal cameras. In dynamic scenarios, the results obtained during the static tests were extended by using more realistic targets: a pedestrian and a car (see Figure 10), either motion or motionless (motionless—protocol (i), motion—protocol (ii), explained below). Targets were used as references to measure the ranges of the thermal cameras concerning different MORs.

**Figure 11:**
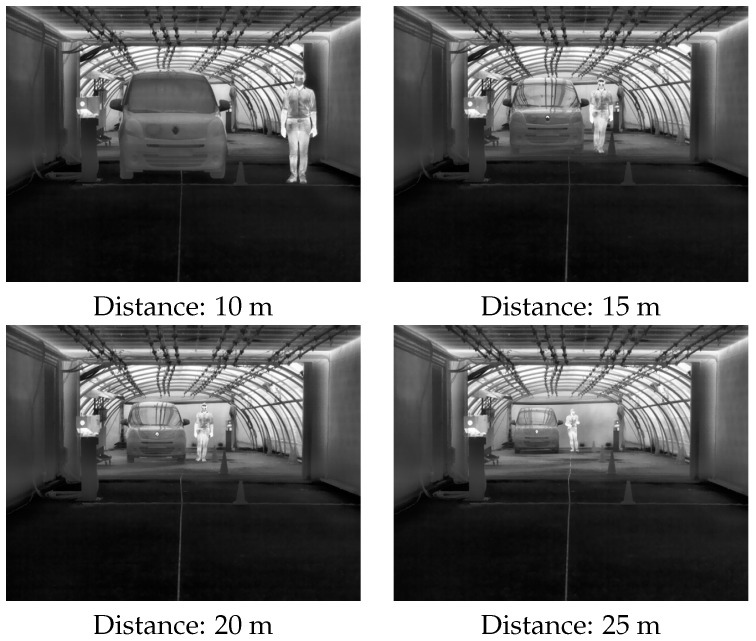
Four different camera-to-target distances used during dynamic scenarios.

As in static scenarios, four different camera-to-target distances were used: 10, 15, 20, and 25 m (see Figure 11).

For dynamic scenarios, two different protocols were used:

(i) The continuous variable MOR varied from 10 to 160 m, alongside a fixed camera-to-target distance. This protocol aimed to analyze the impact on the observability of objects (car and pedestrian) with respect to the camera-to-target distance, considering the presence of fog.
(ii) Car and pedestrian in motion (very low speed) with stable (fix) MOR: Targets made a round-trip journey starting at 25 m away from the cameras and going up to 10 m away from them. This protocol addresses the analysis of the impact of targets’ motion on their observability in the presence of fog.

In both protocols, the car (either motion or motionless) was a parked/stopped electric vehicle, which may be considered a worst-case scenario, since its temperature is close to the ambient temperature. For a moving combustion engine vehicle, it is expected that the heat signature will be more significant. The background in this test can also be considered a worst-case scenario compared to an open-air environment.

**Figure 12:**
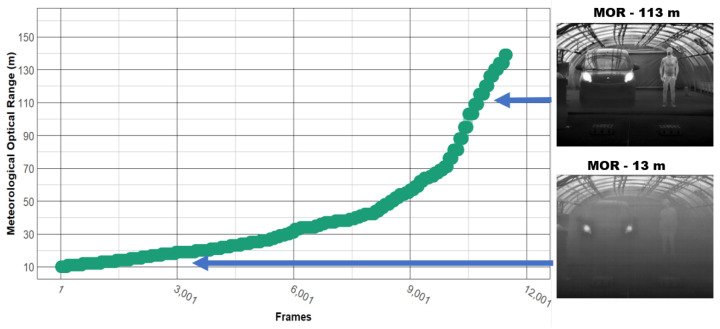
Dynamic scenario, protocol (i): targets at a fixed distance, MOR was varied from 10 to 160 m.

An example of protocol (i) is shown in Figure 12. Note that both the car and the pedestrian remained stationary (at 10, 15, 20 or 25 m), and the MOR (continuous variable) went from 10 to 160 m, just like in the static scenarios.

**Figure 13:**
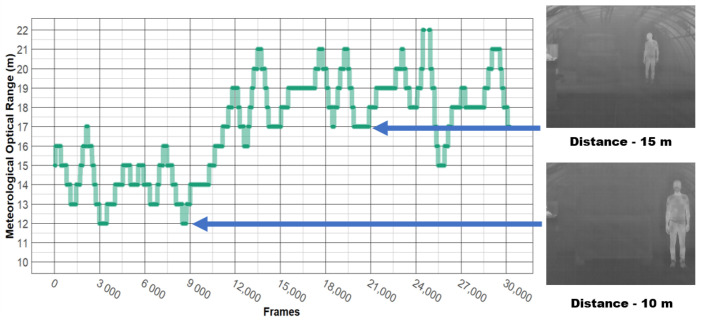
Dynamic scenario, protocol (ii): MOR fixed, targets in motion between 25 and 10 m.

An example of protocol (ii) is illustrated in Figure 13. In this protocol, targets moved at low velocities throughout the test. During that period, the fog density was kept stable. The procedure was the following:

1. Targets started at a distance of 25 m from the camera,
2. After a given time (about one minute), the targets moved until they reached a distance of 10 m from the camera,
3. After a certain time (about one minute), during which the targets were motionless, they returned to their initial positions (25 m from the camera).

## 4. Results

Next, we present the results obtained from the analysis of the data from the static (Section 4.1) and dynamic scenarios (Section 4.2).

### 4.1. Analysis of Static Scenarios

The thermal images that we have processed are grayscale images coded in 8-bit format. In this type of image, the extraction of the intensity in each pixel is very important as an indicator, since it is thanks to these intensity values and the gradients in these intensities that software can detect the contours of objects in the scenes. This was the case for the scenes that were processed, whether for static (fixed targets) or dynamic scenarios (motion or motionless car and pedestrian). Note that in the case of thermal images, light intensity is completely related to temperature, so an analysis of light intensity is equivalent to analyzing the temperature distribution captured by the sensor.

As a side note, the output of the thermal camera is 14 bits wide. Due to the high sensitivity of the camera, to capture the subtle differences in the temperatures of the objects in the scene, the raw pixel data are characterized by a 14 bit range. For visualization purposes, these data were squeezed to 8-bit format, since this is the pixel range that is supported by all display screens. There are numerous techniques to compress a 14-bit histogram to an 8-bit for display purposes. The transformation technique from 14 to 8 bits used by ADASKY is a unique IP, targeting the best experience for the human observer. It is important to emphasize that there is an inevitable loss of information when compressing 14 bits to 8 bits. Hence, the observability results are driven by the ability to distill the relevant details when compressing the data from 14- to 8-bit format and can be improved by specific adjustments to the desired use case, which was not done during this campaign. This means that the results might vary based on the compression method.

Intensity values, and especially intensity changes, were used to define the outlines of objects. In a grayscale image, a sudden change in value characterizes an edge. Such sudden changes in intensities are denominated as contrast [29]. Thus, contrast is an intrinsic property of an image that quantifies the difference in brightness between light and dark parts of it. Contrast characterizes the light distribution of an image (which for thermal images is equivalent to the temperature distribution). Visually, it is possible to interpret it as a spread of the brightness histogram of the image [30]. There are two special cases for a grayscale image:

(a) For zero contrast, the observed image is entirely grey, and therefore, objects (if any) cannot be distinguished from the background.
(b) For maximum contrast, each pixel in the image is either black or white. Then, assuming that objects and the background have different pixel values, easy differentiation of these two elements can be performed.

**Figure 14:**
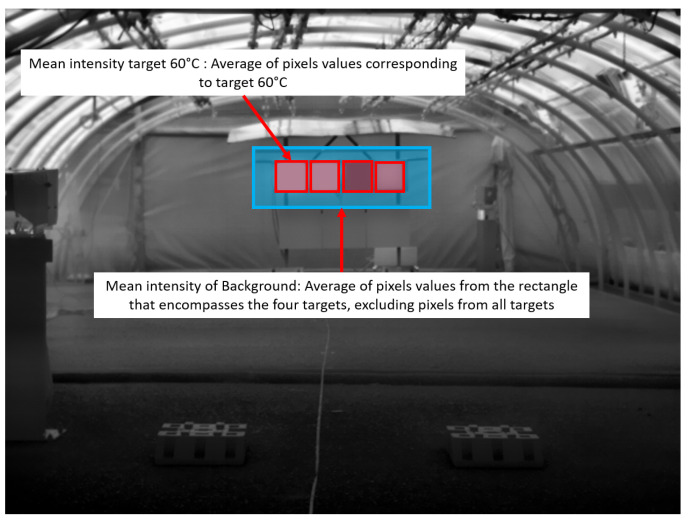
Definition of mean intensity of a target and the background.

These two indicators, intensity and contrast, are intimately linked and are both needed to analyze the content of grayscale images to locate objects in them. For the analysis of data collected from static scenarios, the computation of these two indicators was carried out as follows:

1. The mean intensity in a target is the mean of the intensities (i.e., the values) of the pixels that belong to a target.
2. The normalized global contrast [29] on a target (hereinafter referred to as NGC) is obtained from the normalization of the difference between the mean intensity corresponding to a given target and the background’s mean intensity. In particular, for the static tests, we considered as background the rectangle that encloses the four targets, without considering pixels belonging to the targets (see Figure 14).

Then, the NGC of a given target *T* is computed as follows [29]:

(1) $${\mathrm{NGC}}_{T}=abs\left(\frac{\mathrm{Mean}\;{\mathrm{intensity}}_{T}-\mathrm{Mean}\;{\mathrm{intensity}}_{\mathrm{background}}}{\mathrm{Mean}\;{\mathrm{intensity}}_{T}+\mathrm{Mean}\;{\mathrm{intensity}}_{\mathrm{background}}}\right)$$

Data obtained from the static scenarios allowed the analysis of these two indicators according to four variables:

(a) MOR, whose values ranged continuously from 10 to 160 m.
(b) Temperature; four thermal references: 30, 40, 50, and 60 °C.
(c) Camera-to-target distance; four positions: 10, 15, 20, and 25 m.
(d) AOV; four degrees to evaluate: 18°, 30°, 60°, and 90°.

Next, we present the analysis of the mean intensity and the NGC with respect to the four variables established above.

#### 4.1.1. Analysis of Reference Data

**Figure 15:**
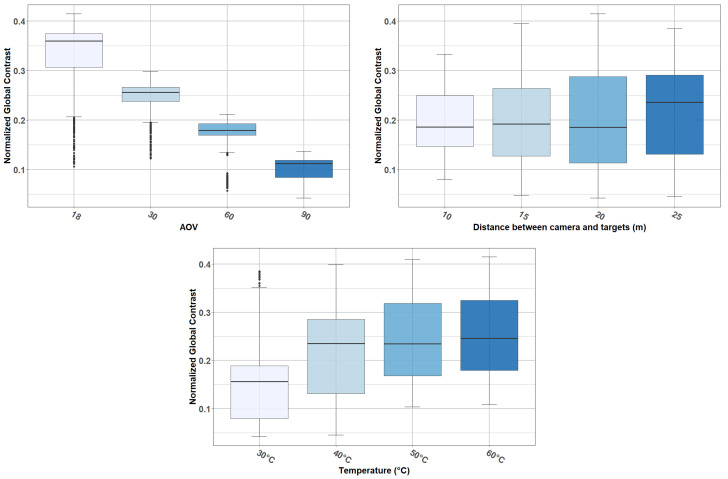
Reference data: contrast distribution according to AOV, camera-to-target distance, and temperature.

We start this analysis by observing the relationships between the different independent variables and the NGC, without the presence of fog. Figure 15 provides boxplots showing the normalized contrast distribution with respect to AOV, camera-to-target distance, and temperature. Such results were obtained from reference data (video sequences without the presence of fog).

From Figure 15, we can observe the following:

- There is an inverse relationship between AOV and contrast (NGC) (left). As Figure 15 shows, there are differences among the NGC means computed from the four AOV (18° (M = 0.32), 30° (M = 0.24), 60° (M = 0.17), and 90° (M = 0.1)). An ANOVA confirmed that these differences are statistically significant and large (F(3, 2120) = 1940.17, *p* < 0.001).
- Even if there is a scattering effect when the camera-to-target distance is increased (right), this variable does not have a large impact on NGC. This was confirmed by an ANOVA, which showed that the effect of the camera-to-target distance is statistically not significant (F(3, 2120) = 2.47, *p* = 0.060).
- Temperature has an impact on the NGC (middle). An ANOVA confirmed that the effect of temperature is statistically significant and large (F(3, 2120) = 118.99, *p* < 0.001).

#### 4.1.2. Analysis of Data with the Presence of Fog

**Figure 16:**
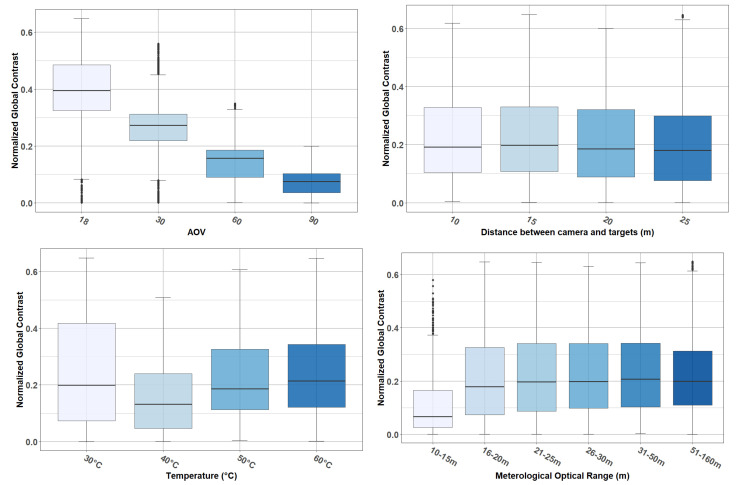
Data collected under foggy weather conditions: contrast distribution with respect to AOV, camera-to-target distance, temperature, and MOR.

**Figure 17:**
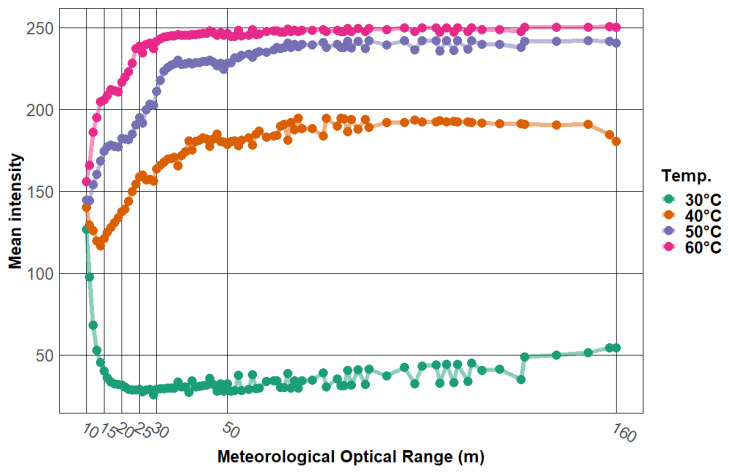
Discretization of the variable MOR into different-sized intervals.

The same study was carried out on the data acquired under foggy situations. Figure 16 shows the contrast distribution with respect to AOV (top left), camera-to-target distance (top right), temperature (bottom left), and MOR (bottom right). Note that for Figure 16, variable MOR was discretized so that it could be presented in boxplot format. This discretization was performed at irregular intervals, since during the analysis of the data, it was observed that most of the changes in mean intensities (and consequently in contrast) occurred at low MOR values (when the fog is denser), as illustrated in Figure 17.

From Figure 16, the following observations are made:

(i) As before, increasing the AOV decreased the contrast level (NGC). Again, an ANOVA confirmed that the effect of AOV was statistically significant and large (F(3, 8040) = 4817.55, *p* < 0.001).
(ii) Unlike when there was no fog, the effect of camera-to-target distance was statistically significant but very small (F(3, 8040) = 16.33, *p* < 0.001).
(iii) The effect of temperature was statistically significant (F(3, 8040) = 182.72, *p* < 0.001). Compared to the previous conditions (no presence of fog), observability of targets at 40°, 50°, and 60 °C was decremented; the target at 40° was the most affected. Contrary to this, the observability of the target at 30 °C was increased. This result can be explained by analyzing Figure 17, which shows how for an MOR equal to 10 m, the mean intensities of all targets collapse to an approximate mean intensity value of 150. This value (150, approximate value) represents the luminous intensity in the image due to fog (image completely saturated by fog). This value is close to the mean intensity of the target at 40 °C, which explains the low contrast between this target and background when there is fog in the scene. Similarly, the increase in contrast (with respect to the scene without fog) in the 30 °C target was due to the differences between the mean intensity of this target and the mean intensity due to the fog.
(iv) The contrast seems to be unaffected when MOR is above fifteen meters. This was confirmed by an ANOVA, from which it was observed that the mean in NGC was between 0.19 and 0.21 for all intervals; MOR was above 15 m. The mean was 0.11 in the interval from 10 to 15 m.

**Figure 18:**
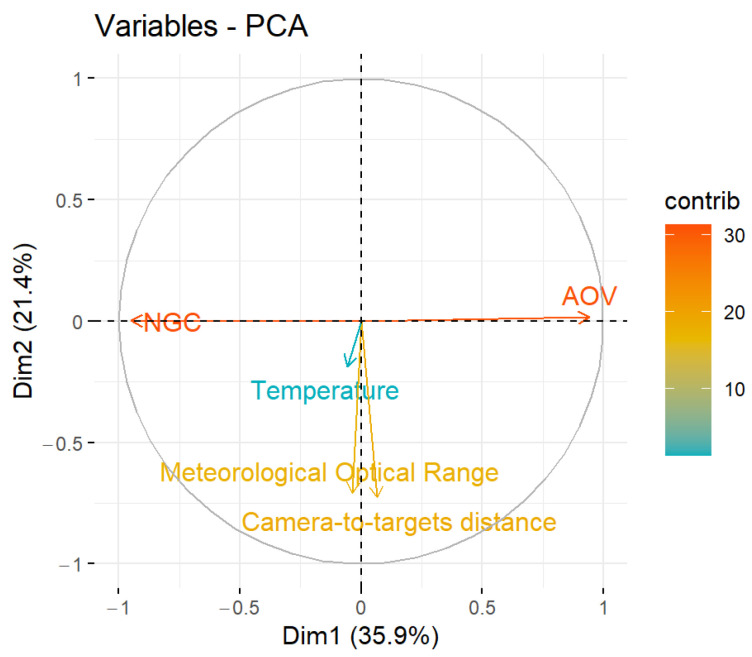
Correlation circle in the PCA.

To deepen in statement (i), principal component analysis (PCA) was carried out using variables: (1) NGC, (2) AOV, (3) temperature (target), (4) camera-to-target distance, and (5) MOR. The results of this study corroborate the negative correlation between the NGC and the AOV (see horizontal axis in Figure 18).

#### 4.1.3. Contrast Attenuation Due to Foggy Conditions

A study of attenuation in contrast caused by MOR is presented next. This parameter quantifies the impact of MOR degradation on the target’s appreciation. Attenuation was calculated per target, distance, AOV, and MOR, and it was estimated as follows:

(2) $$\mathrm{Attenuation}\;\mathrm{in}\;\mathrm{interval}\;A=\left(\left(\frac{\mathrm{NGC}\;\left(\mathrm{median}\right)\;\mathrm{in}\;\mathrm{interval}\;A}{{\mathrm{NGC}}_{\mathrm{reference}}}\right)*100\right)-100$$

Here, interval A denotes a MOR interval (e.g., MOR from 10 to 15 m), and ${\mathrm{NGC}}_{\mathrm{reference}}$ is the median in contrast obtained from reference data (i.e., images taken without the presence of fog). Thus, when referring to the attenuation in the interval of 10 to 15 m, we refer to the difference (in percentage) between the contrast (median) in images taken by an MOR between 10 and 15 m and the contrast (median) in images taken from the reference data. Note that a negative attenuation value indicates loss of contrast (and therefore, loss of target observability) with respect to the reference images. On the contrary, a positive attenuation value indicates that the contrast between the target and the background is greater than in the reference images.

**Figure 19:**
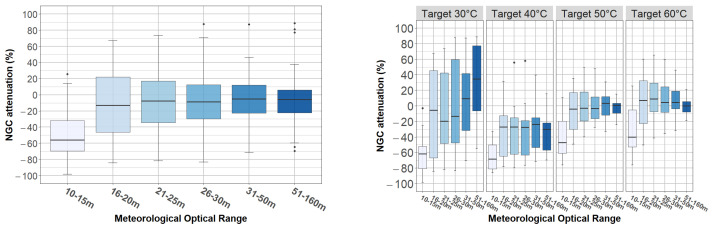
NGC attenuation per MOR and targets.

The results of this analysis are presented in Figure 19. On the one hand, the left image shows the percentage of attenuation for the full dataset. There, we can observe a median in attenuation below 10% when MOR is greater than 20 m, between 10% and 20% when MOR is between 16 and 20 m, and above 50% when MOR is between 10 and 15 m. An ANOVA confirms that the effect of MOR is statistically significant (F(5, 378) = 10.90, *p* < 0.001).

The right side of Figure 19 shows the percentage of attenuation grouped by targets (temperature). Note that for the targets 40°, 50°, and 60 °C, the lower the temperature, the greater the impact of the MOR. For the target at 30 °C, there are even positive attenuation values for when MOR was between 31 and 160 m; however, there was a drastic loss of contrast when MOR went below 16 m. This result agrees with the conclusion presented at point (iii) in Section 4.1.2. Finally, an ANOVA confirmed that: (1) the effect of the temperature was statistically significant and large (F(3, 360) = 41.71, *p* < 0.001), and (2) the interaction between MOR and temperature was significant (F(15, 360) = 3.41, *p* < 0.001).

**Figure 20:**
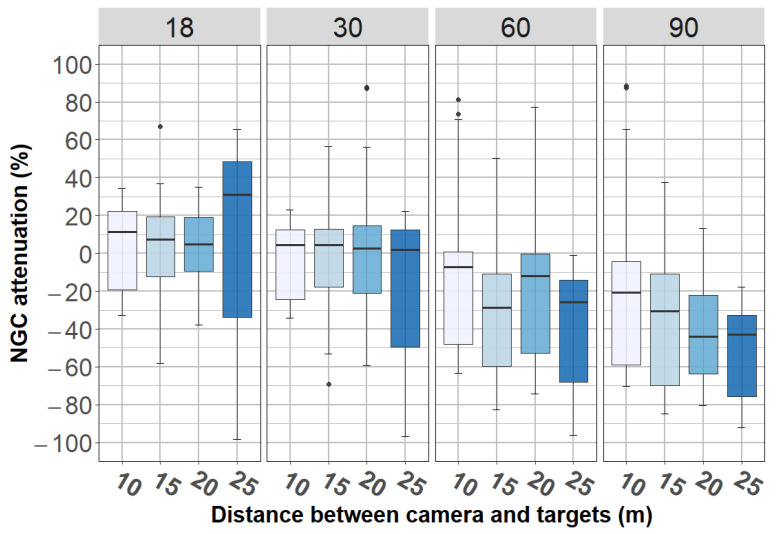
Contrast attenuation according to camera-to-target distance and AOV.

Now, we present in Figure 20 the contrast attenuation according to the distance and AOV. This figure corroborates the inverse relationship between the AOV and the contrast. In addition, by an ANOVA we confirmed that, in the presence of fog, the influence of camera-to-target distance was statistically significant but small (F(3, 376) = 7.15, *p* < 0.001), the effect of the AOV was large (F(1, 312) = 80.13, *p* < 0.001), and the interaction between these two variables was not significant (F(3, 376) = 0.63, *p* = 0.598).

Table 2 summarizes the information presented above. This table shows the percentage of contrast attenuation (quantified with the median) with respect to MOR (columns), grouped by AOV (row) and temperature (row).

In Table 2, it is confirmed that cameras with AOVs of 18° and 30° presented almost no loss of contrast when the MOR was above 15 m; however, once MOR was equal or lower than 15 m, the contrast was drastically affected. The 10 to 15 m interval is of great interest because the limit of the sensors’ operability is linked to strong attenuation in the observability.

#### 4.1.4. Identification of the Operability Limits

**Figure 21:**
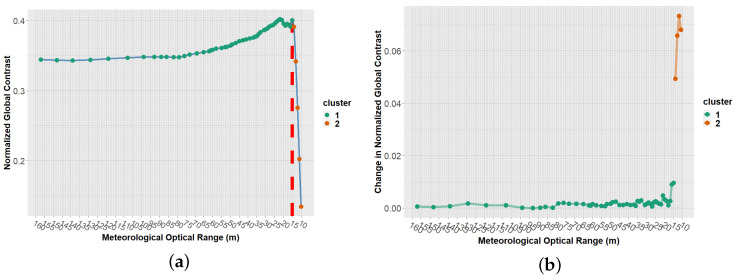
Contrast attenuation and identification of the operational threshold. (**a**) NGC vs. MOR; AOV-18°, Distance-10 m, Target-50 °C; (**b**) Clustering according to change in NGC.

To deepen the previous results, we present Figure 21, which was obtained from the images taken with the AOV 18° camera, with a camera-to-target distance of 10 m. Figure 21 corresponds to the contrast computed from the 50 °C target. The graph on the left presents the relationship between the MOR (horizontal axis) and the NGC (vertical axis). Each point on the graph represents the contrast obtained under a specific MOR. From this graph we can observe a smooth change in NGC when MOR varies between 160 and 16 m; however, once MOR goes below 16 m, the NGC drops drastically. This fact is used to find the limit of operability of the different cameras under the different existing conditions. This limit of operability represents a meteorological optical range from which the contrast between the target and the background is severely affected, making the target’s observability extremely low or even null. Note that the limit of operability does not indicate the MOR at which the target can no longer be distinguished from the background; the operability limit refers to the MOR, from which there is an extreme decrement in the observability of the objects.

The left graph in Figure 21 shows an example of the procedure used to identify the operability limits. This procedure is described below:

1. Select a specific target (temperature), distance, and AOV. For the example presented in Figure 21, the 50 °C target, a camera-to-target distance of 10 m, and an AOV of 18° were selected.
2. From the selected parameters, the relationship between the NGC and the MOR was analyzed (see left side in Figure 21).
3. Going from the highest value of MOR (160 m) to the lowest (10 m), the difference in NGC between successive meteorological optical ranges was computed. From here on, such a difference is referred to as change in NGC. As an example, assuming images obtained under an MOR of 160 and 159 m, the change in the NGC is calculated as follows:
  (3) ChangeinNGC=abs(NGCat160m−NGCat159m)
  The graph on the right in Figure 21 illustrates the computed change in NGC for the selected parameters.
4. Using the change in NGC and hierarchical cluster analysis (HCA) [31], we divided the computed changes into two groups (see right side in Figure 21).
5. Then, by cluster, we computed the means of all elements. Note that each element inside clusters is a value corresponding to a change in NGC.
6. Now, inside the cluster with the lowest mean, we took the lowest MOR. This value corresponds to the limits of operability (in terms of MOR) for the selected parameters (AOV, temperature, camera-to-target distance). For the example in Figure 21, the cluster with the lowest mean was cluster 1. The lowest value of MOR within cluster 1 corresponds to 15 m: below 15 m, the NGC dropped drastically, as the graph on the right of the figure shows.
7. This procedure was repeated for each combination of the parameters target, distance, and AOV.

The results of applying the procedure described above on the complete set of data from foggy weather are summarized in Table 3. This table presents the limits of operability identified for each AOV’s angle, where the median is used as the decisive parameter. Thus, each value in the “Identified Operability *Limit” column represents the medians of the four values from the four camera-to-target distances (10, 15, 20, and 25 m) with respect to a specific AOV and a specific target (temperature).

To summarize the analysis of the static tests, we conclude that for a camera-to-target distance between 10 and 25 m, the observability of objects with a temperature equal or greater than 40 °C is affected by the presence of haze: (1) when MOR is below 16 m for the 18° camera, (2) when MOR is below 15m for the 30° camera, (3) when MOR is below 29 m for the 60° camera, and (4) when MOR is below 37 m for the 90° camera. For the 30° thermal target, results from the 18° and 30° cameras show that the target’s observability is affected when MOR is below 22 m. For the 60° and 90° cameras, the MOR values are 49 and 40 m, respectively. Note that, contrary to the rest of the targets, the threshold of the 60° camera is bigger than the one obtained for the 90° camera. We relate this result to the fact that target’s temperature (30 °C) is close to that of the experiment room (tunnel and greenhouse), which affects the observability of the target regardless of the MOR.

### 4.2. Analysis of Dynamic Scenarios

From the static analysis, the operability limits for the four different cameras (AOV 18°, 30°, 60°, and 90°) were obtained. These limits of operability represent the meteorological optical ranges up to which a negligible impact on contrast is ensured. Below these limits, the NGC drops drastically.

Now, in the dynamic analysis, we seek to expand these results by indicating not only from which value of MOR the contrast is affected, but also from which MOR the objects stop being “detected” by object recognition software. For this, we have decided to use YOLO (You Only Look Once) software, a popular family of object recognition models [9]. As the YOLOv5s pre-trained model is used without any extra training, object recognition is directly linked to the NGC. Thus, the results given in this section are general and independent of the recognition method used.

However, before moving on to the results obtained from the treatment of images with YOLO, a section dedicated to the visual analysis of the images from the dynamic scenarios is presented. This analysis aims to provide a first reference in terms of the MOR at which the targets are no longer detected.

#### 4.2.1. Validation by Human Observers

As a first phase of the dynamic data analysis, a series of visual validations of the videos from the dynamic scenarios were carried out. For this, five people visually verified the videos from protocol (i) (described in Section 3.2) recorded by 18° and 30° cameras, in which a car and a pedestrian were involved. All videos (from 18° and 30° cameras) were distributed among the five volunteers, so each video was verified by a single person. During this process, observers registered the moment at which they saw or no longer saw the objects in the scenario (car/pedestrian). The reason why only the videos from the 18° and 30° cameras were analyzed is because those narrow AOVs can be easier interpreted by a human observer due to higher resolution of the objects in the scene.

**Figure 22:**
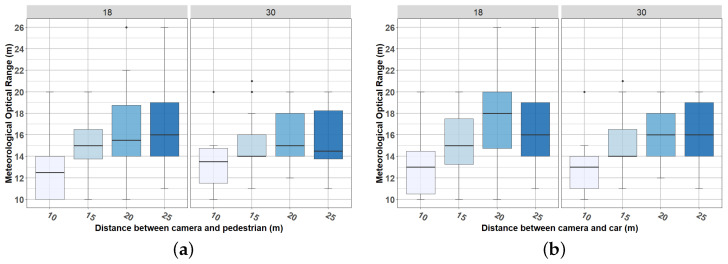
Meteorological optical range at which the car and the pedestrian started to be distinguished by human observers. (**a**) Visual validation—Pedestrian detection; (**b**) Visual validation—Car detection.

Figure 22 summarizes results from visual validations, which shows the MOR at which the targets (car/pedestrian) are distinguished by the participants. The left graph shows results corresponding to the pedestrian, and the right graph shows the results corresponding to the car. Results are grouped by camera-to-target distance (horizontal axis) and by AOV (columns).

Using the median as the decisive parameter, results from the visual validation process were the following:

For the 18° camera:

- With a camera-to-target distance of 10 m, both pedestrian and car were distinguished when the MOR was 13 m (rounded values);
- With a camera-to-target distance of 15 m, both pedestrian and car were distinguished when the MOR was 15 m;
- With a camera-to-target distance of 20 m, the pedestrian was distinguished when the MOR was 16 m, and the car was distinguished when the MOR was 18 m;
- With a camera-to-target distance of 25 m, both pedestrian and car were distinguished when the MOR was 16 m.

For the 30° camera:

- With a camera-to-target distance of 10 m, both pedestrian and car were distinguished when the MOR was 14 m (rounded values);
- With a camera-to-target distance of 15 m, both pedestrian and car were distinguished when the MOR was 14 m;
- With a camera-to-target distance of 20 m, the pedestrian was distinguished when the MOR was 15 m, and the car was distinguished when the MOR was 16 m;
- With a camera-to-target distance of 25 m, the pedestrian was distinguished when the MOR was 15 m (rounded value), and the car was distinguished when the MOR was 16 m.

Observe that both pedestrian and car were found slightly earlier (lower MOR) by the 30° camera (pedestrian: mean camera 18° = 14.875 m, mean camera 30° = 14.25 m; car: mean camera 18° = 15.5 m, mean camera 30° = 14.75 m). This result may have been due to the fact that the 18° camera offered an image very close to the objects, making the pedestrian and car not well appreciated by the observers. As will be presented in the next section, this effect also happened when YOLO was used to measure targets’ observability. It is also noted that the car was detected slightly earlier (lower MOR) than the pedestrian, which we attribute to the size of the object.

#### 4.2.2. Validation Using YOLO

**Figure 23:**
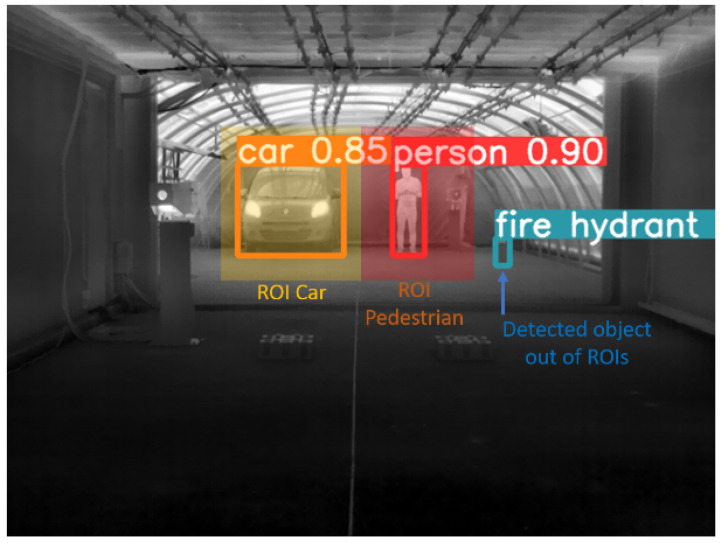
Example of output obtained from YOLOv5 and application of ROIs for discrimination in object detection.

For the analysis of the dynamic scenarios (including targets in motion (protocol (ii) described in Section 3.2 or motionless (protocol (i)) using YOLO software, the following procedure was carried out:

1. Each of the dynamic images sequences was passed through YOLOv5s software using the “yolov5s” model,
2. The outputs from YOLOv5s were: (1) the same image with the bounding boxes that delimit the found/detected objects (see Figure 23), and (2) a semantic classification and a confidence rate corresponding to the objects found. For this study, only the first output was considered. This means that we only considered whether the object was detected or not, regardless of its semantic classification or confidence rate.
3. Thanks to a selection of a region of interest (ROI), the recognition of objects without interest (considered as artefacts) for this study was avoided, limiting the recognition to the car and pedestrian (see Figure 23).

The procedure described above was applied to the full data from the dynamic scenarios (targets in motion (protocol (ii) and motionless (protocol (i)). Table 4 and Table 5 summarize the results obtained from the videos with motionless targets (protocol (i)). Information is displayed in terms of detection rate, which is simply the ratio between the number of images where YOLO detected an object over the total number of images treated. Tables are grouped by MOR (columns), targets (sub columns), camera-to-target distance (rows), and AOV (rows), taking special care in low visibility values (between 10 and 15 m).

From Table 4 and Table 5 we observe a higher detection rate for the pedestrian than for the car. This result is quite interesting because it is directly linked to the temperature of the target: the pixel values of the pedestrian are less variable in the perceived 8-bit image compared to the car, where the heat signature in this case, of an electric vehicle, is spread from the beams. Note that in the case of a combustion engine vehicle, the heat signature is spread from additional various components of the vehicle, which is different from this case.

Considering only the pedestrian, and assuming a detection rate of 90% as the operability limit, we can observe that 18° and 30° cameras were able to operate (i.e., detection rate ≥ 90%) once meteorological visibility was equal or greater than 13 and 14 m, respectively. The 60° camera showed an operability limit of 16 m, which is much better than expected if we refer to the static analysis. In the case of the 90° camera, detection rates below the operability limit were obtained in all the cases.

Similarly, considering only the car, and assuming a detection rate of 90% as the operability limit, the 18° camera showed a limit of 31 m, the 30° camera showed a limit of 21 m, and the 60° camera showed a limit of 31 m. Again, for the 90° camera, detection rates below the operability limit were obtained in all the cases. Note that car detection was achieved at a lower MOR level when using images from the 30° camera rather than from the 18° camera. We attribute this phenomenon to the fact that the silhouette of the car is not fully captured when a small AOV is used, which makes the detection task difficult.

**Figure 24:**
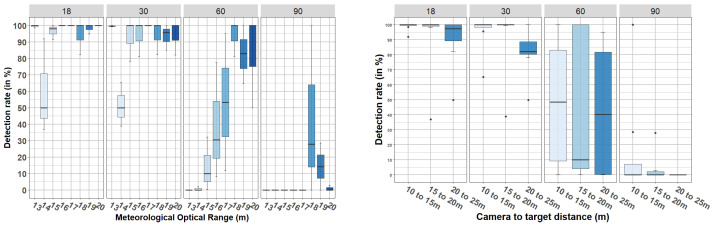
Detection rate (in %) obtained during dynamic scenarios—targets in motion—pedestrian detection.

**Figure 25:**
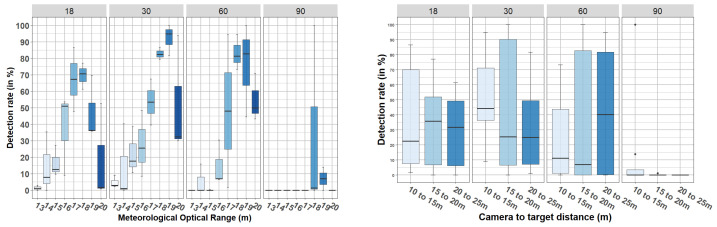
Detection rate (in %) obtained during dynamic scenarios—targets in motion—car detection.

The results from videos with targets in motion (protocol (ii)) are presented in Figure 24 and Figure 25. Figure 24 shows the results from pedestrian detection, and Figure 25 shows the detection rate of the car. In each of these figures, two graphs are shown: the graph on the left shows the detection rate (vertical axis) with respect to the MOR (horizontal axis), and the graph on the right shows the detection rate (vertical axis) with respect to the camera-to-target distance (horizontal axis). In both cases, the data are grouped by AOV (vertical columns). Observe that the camera-to-target distance has been discretized into three ranges: 10 to 15, 15 to 20, and 20 to 25 m. This discretization was due to the motion of the target.

From these figures we can observe that, with both the 18° and 30° cameras, there was a pedestrian detection rate ≥ 90% from 15 m MOR, which complements the results presented in Table 4 and Table 5. In addition, the right graph in Figure 24 shows that considering all data with MORs between 13 and 20 m, for the 18° camera, there was a median greater than 90% in pedestrian detection for a camera-to-target distances between 10 and 25 m. This was not the case for the 30° camera, for which there was a detection rate close to 80% when the camera-to-target distance was between 20 and 25 m. Regarding the car, in Figure 25 it is shown that the detection rates were lower than the defined operability limit (90%), corroborating the previous results presented in Table 4 and Table 5.

Finally, we can observe that the detection of both objects (pedestrian and car) was much better in images captured with the cameras with AOVs of 18° and 30° than those captured with cameras with AOVs of 60° and 90°, confirming the results from static tests. As shown in Section 4.2.1, Figure 24 and Figure 25 show that the car was better detected by the 30° camera than by the 18° camera (contrary to what happened with the pedestrian). As in Section 4.2.1, we attribute this to the fact that the edges of the car are not well highlighted when small AOVs are used (the silhouette of the car is not fully captured). Finally, ANOVA confirmed that the interaction between the AOV and the camera-to-target distance was statistically not significant for the pedestrian (F(6, 79) = 0.07, *p* = 0.998) or the car (F(6, 79) = 0.62, *p* = 0.715).

## 5. General Summary of Results with Identification of Borderline Cases

Throughout this research, we analyzed the performance responses of a set of thermal sensors under the influence of extreme foggy weather. To be more specific, we evaluated the thermal cameras under increasingly thick fog. Then, we found the breaking point, i.e., the very degraded situation beyond which the thermal sensor becomes unusable. This research allowed us to better characterize the video sensor used, which is one of the AWARD project’s main goals.

For this, two types of scenarios were used: (1) static, where motionless targets with different temperatures placed at specific distances from the sensors were used; (2) dynamic, where an electric car and a pedestrian were used as moving and motionless targets. In both cases, four thermal cameras with different angles of view (AOVs) were used, which allowed the analysis of this variable with respect to fog intensity.

In static scenarios, the operability limits for the four tested cameras (AOV 18°, 30°, 60°, and 90°) were obtained. These limits represent meteorological optical values at which the contrast between the objects of study and the background is severely affected. Above these limits, a negligible impact on contrast due to MOR is ensured. Below these limits, the contrast drops drastically, making any object’s observability extremely low.

Thus, the results from static scenarios show that, using cameras with AOV 18° and 30°, observability of 40, 50, and 60 °C targets is severely affected when the MOR is lower than 16 or 15 m, respectively. In the case of cameras with AOVs of 60° or 90°, for the same targets, it was found that observability is affected when the MOR is below 29 or 37 m, respectively.

Four interesting conclusions resulting from the analysis of static scenarios were obtained: (1) the angle of view (AOV) has an inverse relationship with the contrast level: increasing the AOV increases the impact of MOR on the observability of the objects, which means that it decreases the contrast (NGC); (2) the influence of the temperature on the objects’ observability is large (i.e., the interaction between MOR and temperature is large), as expected; (3) for the range of distances used in these scenarios, the influence of the camera-to-target distance in the observability is small; and (4) the interaction between AOV and camera-to-target distance is not statistically significant.

For dynamic scenarios, we extended these results by indicating not only from which value of MOR the contrast is affected, but also from which MOR value the objects stop being “detected” by both human observers and object recognition software.

For the first part, five people visually verified the 8-bit videos in which a car and a pedestrian were involved. During this process, observers registered the moment at which they started or stopped to see any of the targets present in the scenario (car/pedestrian). From this analysis, we concluded that, with a camera-to-target distance between 10 and 25 m, and using the mean (rounded value) as a decisive parameter, the observers were able to distinguish: (1) the pedestrian in the 18° camera when MOR ≥ 15 m; (2) the pedestrian in the 30° camera when MOR ≥ 14 m; (3) the car in the 18° camera when MOR ≥ 15 m; and (4) the car in the 30° camera when MOR ≥ 15 m.

Finally, for validation using object recognition software, we decided to use YOLO (You Only Look Once). As the YOLOv5s pre-trained model was used without any extra training, object recognition was directly linked to the NGC. Thus, the results given in this section are general and independent of the recognition method used.

Then, the results from dynamic scenarios showed that in general the pedestrian was better detected than the car. This fact is quite interesting because it is directly linked to the temperature of the target. Likewise, it was observed that the car was better detected by the 30° and 60° cameras than by the 18° camera. We attribute this to the fact that the edges of the car are not well highlighted when small AOVs are used. To conclude, we observed that considering only the pedestrian, and assuming a detection rate of 90% as the operability limit, 18° and 30° cameras were able to operate without degradation as long as MOR was equal to or greater than 13 or 14 m, respectively. In the case of the 60° camera, it showed an operability limit of 16 m, which is much better than expected after static analysis. In the case of the 90° camera, detection rates below the operability limit established were obtained in all the cases.

Finally, it is important to emphasize that the performance of YOLO can be improved if the model is trained taking into account the thermal data. An interesting particular case would be the use of the uncompressed 14-bit images for both model training and object detection. This idea is exploited in ADASKY’s AI perception layer. However, this product is not part of the AWARD program and thus was not part of this research.

## 6. Conclusions and Perspectives

The objective of this research was to analyze the resilience of thermal sensors in very unfavorable fog conditions. In the introduction, we stated that the goal in terms of transport is to be able to exploit these thermal sensors in order to complement or replace other video sensors which could not correctly detect obstacles in foggy conditions. Indeed, one of the final goals of the AWARD project is to develop a series of sensors able to operate 24/7, even under harsh weather conditions. We have shown here that, at a distance between 10 and 25 m away, with the analyzed thermal cameras and a dedicated program, one can detect objects under very thick fog conditions (up to a meteorological optical range of 13 m in the best case). This is very encouraging for the applications involving autonomous vehicles.

Thus, the threshold of 90% detection was a minimum reached, and at very low distances to obstacles (less than 15 m). In principle, at that distance and depending on the autonomous vehicle’s speed, the breaking distance is very short; but detection is effective at that distance even with very low visibility. Furthermore, in the various graphs displayed, we can see that as soon as we consider a meteorological optical range of more than 16 m, the detection rate rapidly moves toward 100%. In a very safety-critical context, which is the case for autonomous vehicles, it is important to be close to 100%: in this kind of application, misdetection could result in collisions. We also showed that the thermal images are compatible with detection and recognition algorithms based on artificial intelligence and neural networks. The next step will consist in testing these thermal sensors on real cases, that is to say, installed on an autonomous vehicle and circulating on road infrastructure. In addition to this, for quantifying YOLO detection, the detection rate was used as a parameter (the total number of frames with some detection over the total number of frames). As an extension of this research, we propose to measure not only the number of detections but also their intermittency—e.g., how many detections are there in a one-second video? This will allow a better understanding of the operability limits regarding the use of detection software.

The global results are very encouraging. However, a more intensive evaluation is needed to have definitive conclusions on the resilience of the sensor in the face of extreme foggy situations: more scenarios, with more static or moving objects. A more extensive evaluation plan is also needed. In terms of processing, there are several possibilities: either we use ADASKY’s proprietary software and test the hardware+software combination in foggy conditions, or we continue to use YOLO but by enriching the learning base with thermal images because the COCO base currently used by YOLO does not contain any. Furthermore, the thermal camera’s configuration can also be tuned to improve observability for the use-cases and conditions tested.

## Figures and Tables

**Table 1:** The ADASKY camera’s key technical specifications.

Category	Attribute	Specification
Image	Sensor Technology	Uncooled VOx microbolometer
	Resolution	VGA (640 × 480)
	Spectral Responsivity	7.5–13.5 µm
	NETD (@ 25 °C F/#1, 30 Hz)	<50 mK
Optics	Focus range	10 m~∞ (Optimized to 100 m)
	MTF	0.20 @ Cutoff
	Thermal Compensation	Athermal
ISP	ASIC	ADA1 ISP ASIC
		Technology: 28FDSOI
		Compliance: AEC-Q100 grade 2 compliance ready
		ASIL B compliance ready
	Frame rate	8.4 Hz/30 Hz
		Optional—60 Hz
	Video Out format	14 bit grayscale optimized for computer vision
		8 bit grayscale optimized for preview
	Latency (pixel acquisition to output time)	<2 ms (computer vision only mode 14 bit)
	Nonuniformity correction	Shutterless
	Frame sync	Producer mode—Internal free running
		Consumer mode—External sync based
Power	Power supply	6–19 V power over Coax
	Power consumption	~1.1 W typical, 1.5 W max
Lens Heater	Power consumption	3.5 W
Environmental	Operating condition	−40~+85 °C Operational mode
		−40~+105 °C Storage temperature
	Waterproof	IP67, IP69K on front lens
Physical	Dimension (WLP)	⌀26 mm × 39 mm 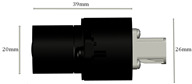
	Weight	≤55 g

**Table 2:** NGC attenuation (in %) with respect to MOR, AOV, and temperature.

Median in NGC Attenuation (in %)							
**AOV**	**Temperature**	**Meteorological Optical Range**					
		**10–15 m**	**16–20 m**	**21–25 m**	**26–30 m**	**31–50 m**	**51–160 m**
18	30 °C	−45	70	117	154	157	173
	40 °C	−48	0	6	3	0	−13
	50 °C	−19	23	27	22	18	7
	60 °C	−3	40	41	34	26	11
30	30 °C	−63	31	71	85	100	105
	40 °C	−50	−12	−15	−18	−15	−21
	50 °C	−24	12	10	9	8	4
	60 °C	−10	21	19	16	12	4
60	30 °C	−64	1	33	51	69	85
	40 °C	−76	−53	−52	−53	−43	−46
	50 °C	−52	−25	−25	−11	−8	−8
	60 °C	−45	−13	−4	−3	−1	−4
90	30 °C	−74	−35	−17	−12	4	21
	40 °C	−80	−63	−64	−59	−53	−55
	50 °C	−62	−42	−30	−21	−17	−13
	60 °C	−61	−39	−26	−23	−15	−14

**Table 3:** Identification of operability limits.

AOV (°)	Target (Temperature)	Identified Operability Limit (MOR in Meters)
18	30 °C	22
	40 °C	16
	50 °C	16
	60 °C	15
30	30 °C	22
	40 °C	15
	50 °C	15
	60 °C	15
60	30 °C	49
	40 °C	29
	50 °C	25
	60 °C	20
90	30 °C	40
	40 °C	37
	50 °C	28
	60 °C	24

**Table 4:** Detection rates obtained from YOLO. MOR from 10 m to 15 m.

Detection Rate (in %)													
AOV (°)	Distance (m)	Meteorological Optical Range											
		10 m		11 m		12 m		13 m		14 m		15 m	
		Ped	Car	Ped	Car	Ped	Car	Ped	Car	Ped	Car	Ped	Car
18	10	22	0	25	0	59	19	91	37	96	34	98	22
	15	4	0	42	0	76	0	100	8	100	51	100	34
	20	3	0	63	0	100	0	100	10	100	58	100	94
	25	0	0	45	0	100	2	100	48	100	98	100	100
30	10	10	0	57	0	58	10	57	89	100	100	100	100
	15	0	0	15	0	29	0	96	2	100	26	100	14
	20	0	0	25	0	84	0	100	20	100	82	100	92
	25	0	0	22	0	56	1	100	3	100	50	100	58
60	10	0	0	5	1	40	0	51	1	77	1	97	0
	15	0	0	0	0	0	0	2	0	10	0	52	9
	20	0	0	0	0	2	0	44	5	73	3	92	23
	25	0	0	2	2	5	3	58	7	91	3	100	21
90	10	0	0	0	0	0	0	12	0	8	0	15	0
	15	0	0	0	0	0	0	0	0	0	0	0	0
	20	0	0	0	0	0	0	0	0	0	0	0	0
	25	0	0	0	0	0	0	0	0	2	0	11	0

**Table 5:** Detection rates obtained from YOLO. MOR from 16 m to 160 m.

Detection Rate (in %)									
AOV (°)	Distance (m)	Meteorological Optical Range							
		16–20 m		21–30 m		31–50 m		51–160 m	
		Ped	Car	Ped	Car	Ped	Car	Ped	Car
18	10	100	29	100	59	100	99	100	100
	15	100	71	100	96	100	100	100	100
	20	100	100	100	100	100	100	100	100
	25	100	100	100	100	100	100	100	100
30	10	100	100	100	100	99	100	100	100
	15	100	70	100	100	100	100	100	100
	20	100	100	100	100	100	100	100	100
	25	100	98	100	100	100	100	100	100
60	10	99	14	100	82	99	100	100	100
	15	96	61	99	90	100	100	92	100
	20	100	92	100	100	100	100	100	100
	25	100	60	100	97	100	100	100	100
90	10	26	16	49	91	56	100	90	100
	15	0	0	1	1	4	5	30	37
	20	0	0	51	19	100	100	100	100
	25	46	0	46	0	46	0	46	41

## Data Availability

Not applicable.
